# Evaluation of cross-protection of a reduced-dose PRRS MLV vaccine against the NADC30-like PRRSV challenge

**DOI:** 10.3389/fvets.2024.1492173

**Published:** 2024-11-13

**Authors:** Jiayu Liu, Xinyu Yan, Wensi Wu, Yan Li, Shuaibin Xing, Shan Zhao, Xiaobo Huang, Qin Zhao, Yiping Wen, Sanjie Cao, Senyan Du, Qigui Yan, Nanfang Zeng

**Affiliations:** ^1^Giantstar Farming & Husbandry Co., Ltd, Chengdu, Sichuan, China; ^2^College of Veterinary Medicine, Sichuan Agricultural University, Chengdu, Sichuan, China

**Keywords:** MLV vaccine, NADC30-like PRRSV, vaccine, efficacy, pathogenicity

## Abstract

**Introduction:**

At present, the NADC30-like strain has become the prevalent strain of PRRSV in China. Many studies have found that existing commercial vaccines are ineffective or provide only limited protection. No study has investigated the cross-protection of different dosages of commercial MLV vaccines against NADC30-like PRRSV. Therefore, this study assessed the effectiveness of various dosages against a NADC30-like PRRSV infection using commercial PRRSV vaccines, Ingelvac PRRS MLV, which have been widely utilized in China.

**Methods:**

In this study, we immunized piglets with four different dosages of the MLV vaccine and infected piglets within a nasal way with NADC30-like CF PRRSV at 28 days post-vaccination. We observed the status of pigs before and after the challenge of NADC30-like PRRSV CF strain and reflected the protective effect of different dosages of MLV vaccine through multiple assays.

**Results:**

Compared to those piglets immunized with 1 dosage, the piglets immunized with 0.01 dosage had better performance, such as the highest average daily gain before the challenge, lesser lesions and viremia after the challenge, low clinical score, and stable temperature during the study. However, the piglets immunized with 0.01 dosage still showed viremia, viruses were detected in their lungs, tonsils, and inguinal lymph nodes, and pathological lesions occurred in their lung. Immunohistochemistry staining of the lung of vaccinated piglets revealed a similar viral load to that of unvaccinated piglets, suggesting that immunization could not completely remove the virus from the vaccinated piglets’ tissues.

**Discussion:**

Our research suggests that the MLV vaccine could provide limited protection against the NADC30-like PRRSV infection, and lowering the dosage to 0.01 may produce better protective efficacy. In the context of identifying the immunological target, comprehending the virulence of the virus in the field, and guaranteeing safety, we might be able to reevaluate vaccination dosages to achieve higher economic value.

## 1 Introduction

Porcine reproductive and respiratory syndrome (PRRS) is the number one killer affecting the pig industry, apart from African swine fever (1, 2). The etiological agent of PRRS is the porcine reproductive and respiratory syndrome virus (PRRSV), which belongs to the order Nidovirales, family Arteriviridae (3). Since its first discovery in 1996, PRRSV has been prevalent in China for approximately 30 years. Porcine reproductive and respiratory syndrome virus (PRRSV) is a small, enveloped, single-stranded positive-sense RNA virus. According to the latest classification, PRRSV was classified into the genus Porartevirus, family Arteriviridae, and order Nidovirales (4). Based on phylogenetic research and the features of the virus’s genome, PRRSV with two genotypes may be further classified into several subgenotypes (5). Lelystad and VR2332 are the representative strains of PRRSV’s European genotype 1 and North American genotype 2, respectively (6).

In 2001, a PRRSV variant strain, MN184, was isolated from sows in the United States (7). Whole-gene sequence analysis revealed 131 discontinuous amino acid deletions in the nsp2 region of the strain, including a 111-aa deletion at position 322–432, a 1-aa deletion at position 483, and a 19-aa deletion at position 504–522. In 2008, a highly virulent PRRSV-2 strain with the same characteristics was reported in Iowa, USA, namely the NADC30 strain, associated with severe respiratory diseases (8). Since 2013, China has reported several strains of the virus similar to NADC30 nucleotides, named NADC30-like PRRSV, and its prevalence has been increasingly extensive, emerging in over 13 provinces or regions (9). The detection rate of NADC30-like PRRSV has been gradually increasing and has surpassed HP-PRRSV as the main circulating strain in some areas of China since 2016 (10, 11). For example, testing of clinical samples from Henan Province in central China found that 83.3% of the isolates belonged to the NADC30-like strain (12). China has extensively used five commercial PRRSV vaccines so far, which use the matching virus strains: JXA1-P80 (lineage 8), HuN4-F112 (lineage 8), GDr180 (lineage 8), TJM-F92 (lineage 8), and VR2332 (lineage 5) (12). Among them, the most commonly used is Ingelvac PRRS MLV (13). Currently, most commercially available vaccines only provide partial protection and symptom relief against NADC30-like strains but cannot achieve full immunity (14), and enterprises spend a lot of money on them. Ingelvac PRRS MLV strain belongs to lineage 5 virus, whereas NADC30-like PRRSV belongs to lineage 1 according to the global PRRSV classification systems (6). According to our investigation, the vaccine dosage has been generally reduced in the clinical pig industry, from 1 dosage (10^4.8^ TCID50) to 0.5 dosage (5 × 10^3.8^ TCID50). From the perspective of immunological mechanisms, the traditional concept of vaccine dose is often a fixed value determined by a large number of clinical trials. Considering the appropriate reduction of vaccine doses challenges this established understanding. Previous studies of vaccine use have focused on the preventive effect of the disease, and relatively little consideration has been given to the cost of vaccine use. The study on reducing vaccine use is a comprehensive analysis and evaluation of the costs and benefits of vaccine use from the perspective of economic benefits. Thus, in this study, a NADC30-like PRRSV challenge at 35 days post-vaccination (dpv) was used to assess the protective effectiveness of various dosages of Ingelvac PRRS MLV in vaccinated piglets.

## 2 Materials and methods

### 2.1 Virus, cells, and MLV vaccine

The Ingelvac PRRS MLV vaccine, purchased from Boehringer Ingelheim, Germany, is one commercial live modified PRRSV vaccine that includes the virus VR2332 strain. The MLV vaccine was diluted to various dosages: 1 dosage (10^4.8^ TCID50), 0.5 dosage (5 × 10^3.8^ TCID50), 0.1 dosage (10^3.8^ TCID50), and 0.01 dosage (10^2.8^ TCID50), with vaccination procedures conducted according to the manufacturer’s guidelines. The viral strain used in this study was *CF*, an isolated NADC30-like PRRSV provided by Chengdu SG-Biotech Co., Ltd.

### 2.2 Animal trials for vaccination and challenge

A total of 35 3-week-old piglets that were free of the porcine circovirus 2 (PCV2), classical swine fever virus (CSFV), pseudorabies virus (PRV), and PRRSV were randomly assigned to seven groups, each consisting of five piglets. They were intramuscularly (IM) inoculated with phosphate-buffered saline (PBS) and 1 dosage, 0.5 dosage, 0.1 dosage, and 0.01 dosage of vaccine. Thirty-five days after vaccination, piglets were intranasally challenged (2 mL in total). One mock and each of the vaccinated groups was challenged with a 10^5^ TCID50/mL dosage of *CF* PRRSV. The rectal body temperatures and clinical signs of the piglets were recorded once every 2 days throughout the experiment, and body weight was measured every week. During the experiment, the status of the piglets was recorded by scoring, including gross clinical scores (GCSs), respiratory clinical scores (RCSs), and nervous signs scores (NSSs). The specific scoring rules are shown in Table 1. All piglets were humanly euthanized at 28 days post-challenge (dpc).

**Table 1:** Clinical sign scoring system used for infected pigs.

	Symptom types	Evaluation criterion	Score
Gross clinical scores, GCS	Temperature	T ≤ 39.9°C	0
		40.0°C ≤ T ≤ 40.9°C	1
		41.0°C ≤ T	2
	Appetite	Normal	0
		Loss of appetite	1
	Mentality	Normal	0
		Unclear consciousness/Drowsiness	1
	Skin	Normal	0
		Cyanochroia	1
Respiratory clinical scores, RCS	Respiratory disease	Normal	0
		Rapid breathing during tension	1
		Rapid breathing during rest	2
		Rapid breathing and difficulty breathing during rest	3
		Severe shortness of breath, difficulty breathing, irregular breathing, and difficulty breathing	4
	Cough	Normal	0
		Cough	1
	Runny nose	Normal	0
		Runny nose	1
Nervous signs scores, NSS	Neurological symptoms	Normal	0
		Tremble	1
		Ataxia	2
		Arm pull	3
		Paralysis	4

* Usual condition: total scores = GCS + RCS + NSS; If piglet died: total scores GCS + RCS + NSS + 5; 0 ≤ total scores ≤ 20.

### 2.3 Serology and viremia test

Piglets’ blood was drawn from the anterior vena cava every week after immunization and at 7-, 14-, 21-, and 28-day post-vaccination to identify viremia and specific antibodies to PRRSV. After the blood sample is collected, it is left at room temperature for 1–2 h to allow it to solidify naturally and precipitate serum. The extracted serum was divided into dry and clean aseptic centrifuge tubes, labeled, and placed in an ultra-low temperature refrigerator at −80°C. The IDEXX PRRS 2XR Porcine Reproductive & Respiratory Syndrome Virus Antibody Test Kit (IDEXX Laboratories) was used as directed by the manufacturer to assess PRRSV-specific ELISA antibody titers. S/P ratios were used to report PRRSV-specific antibody titers, and serum samples were deemed positive if the S/P ratio was 0.4 or greater.

Total RNA was extracted from serum samples by using the Virus DNA/RNA Extraction Kit 2.0 (prepackaged) (Vazyme) and stored in the −80°C ultra-low temperature refrigerator. Using the HiScript II U+ One Step qRT-PCR Probe Kit (Vazyme), real-time PCR was conducted using the cDNA from each sample. According to the conserved sequence of the M gene, q-PCR primers and the probe were designed using AlleleID 6.0 software. The primers of real-time PCR were PRRSV M-F: 5’-CACTACGGTCAACGGCACATT-3′; PRRSV M-R: 5’-GCATATTTGACAAGGTTTACCACTCC-3′. The TaqMan probe was synthesized as FAM-CTTTTCTGCCACCCACACGAGGCTT-DBQ. The conditions for amplification were 55°C for 15 min and 95°C for 30 s, followed by 45 cycles of 95°C for 10 s and 60°C for 30 s. A standard curve was generated with 10-fold serially diluted plasmid standards of 10^1^–10^9^ copies/μL. The viral load of each sample was calculated using the standard curve equation constructed previously (*y* = 45.46–3.32x, *R*^2^ = 0.996).

### 2.4 Histopathology and immunohistochemistry examination

**Figure 1:**
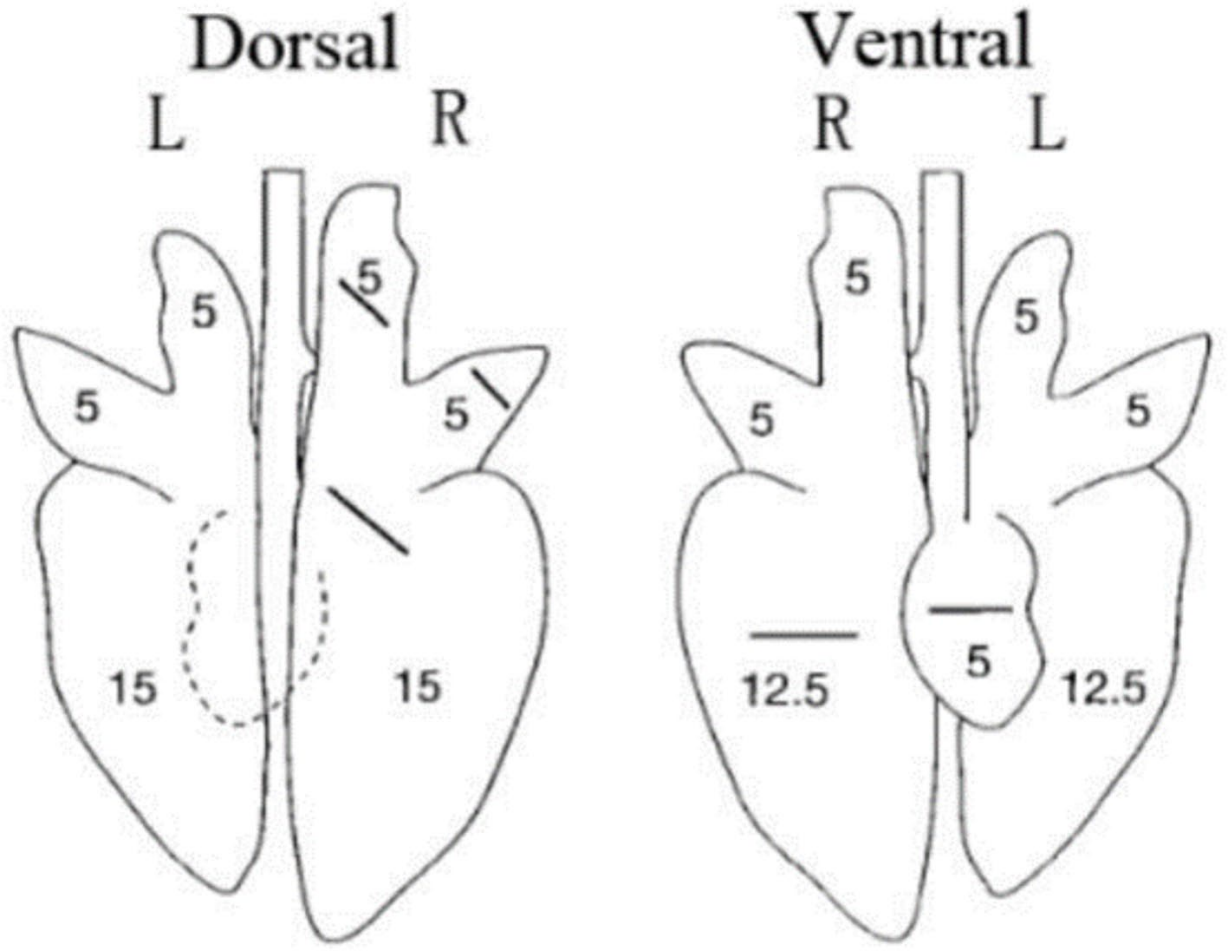
Scoring criteria for gross lesions in pulmonary autopsy.

All piglets were humanly euthanized at 28 dpc. At necropsy, the three parts of the lung were fixed in 10% buffered neutral formalin for hematoxylin and eosin (H&E) and immunohistochemistry staining. Photos taken with a 200× microscope were used to visualize the slides. The lungs were observed, and the lesions were recorded and scored according to Figure 1 and Table 2.

**Table 2:** Scoring criteria for lung tissue section.

Score	Pathological condition
0	No obvious lesions
1	Slight pathological changes, thickening of the alveolar wall, infiltration of inflammatory cells, stasis, or slight shedding of mucosal epithelial cells
2	Interstitial pneumonia and slight focal distribution
3	Interstitial pneumonia, moderate diffuse distribution, or severe focal distribution, more than 2/5 of the lesion area
4	Interstitial pneumonia, severe diffuse distribution, and pathological tissue area accounted for more than 4/5

### 2.5 Statistical analysis

All data were expressed as the mean value of 5 piglets ± SEM. Using the GraphPad Prism 9 program (San Diego, CA), statistical analyses were carried out by performing two-way ANOVA and then Tukey’s *t*-test. When *p* < 0.05, differences were deemed statistically significant.

## 3 Results

### 3.1 Clinical presentation and piglet growth performance

All data were expressed as the mean value of five piglets ± SEM. Using the GraphPad Prism 9 program (San Diego, CA), statistical analyses were carried out by performing two-way ANOVA and then Tukey’s *t*-test. When *p* < 0.05, differences were deemed statistically significant.

**Figure 2:**
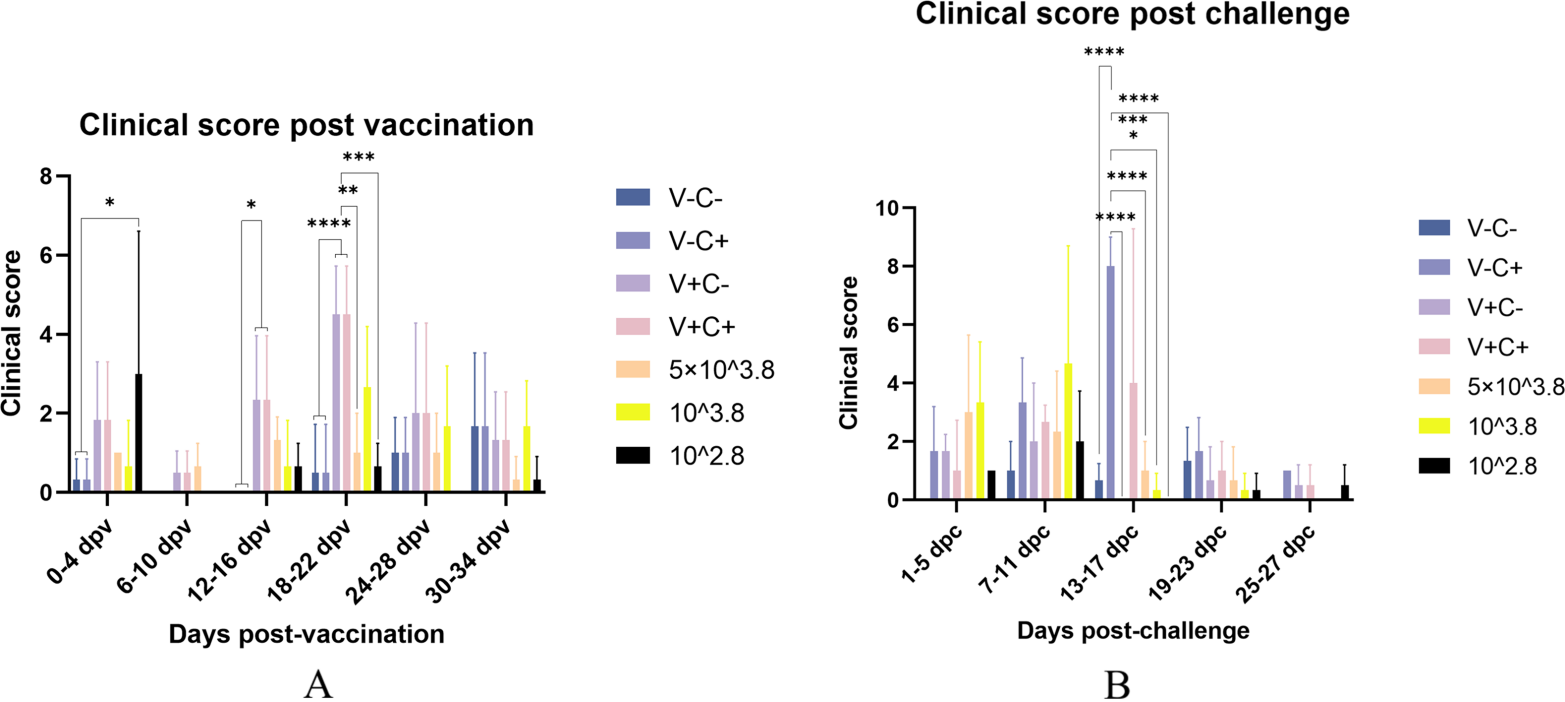
Clinical scores evaluated according to Table 1. The clinical scores of the piglets were recorded once every 2 days and compared every adjacent three counts. (A) Clinical score post-vaccination on different dates. (B) Clinical score post-challenge on different dates. V ± means groups with the vaccination or not. C ± means groups with the challenge or not. All the reduced-dose groups were challenged. * Indicates a statistically significant difference (*: *p* < 0.05; **: *p* < 0.01; ***: *p* < 0.001; ****: *p* < 0.0001).

**Figure 3:**
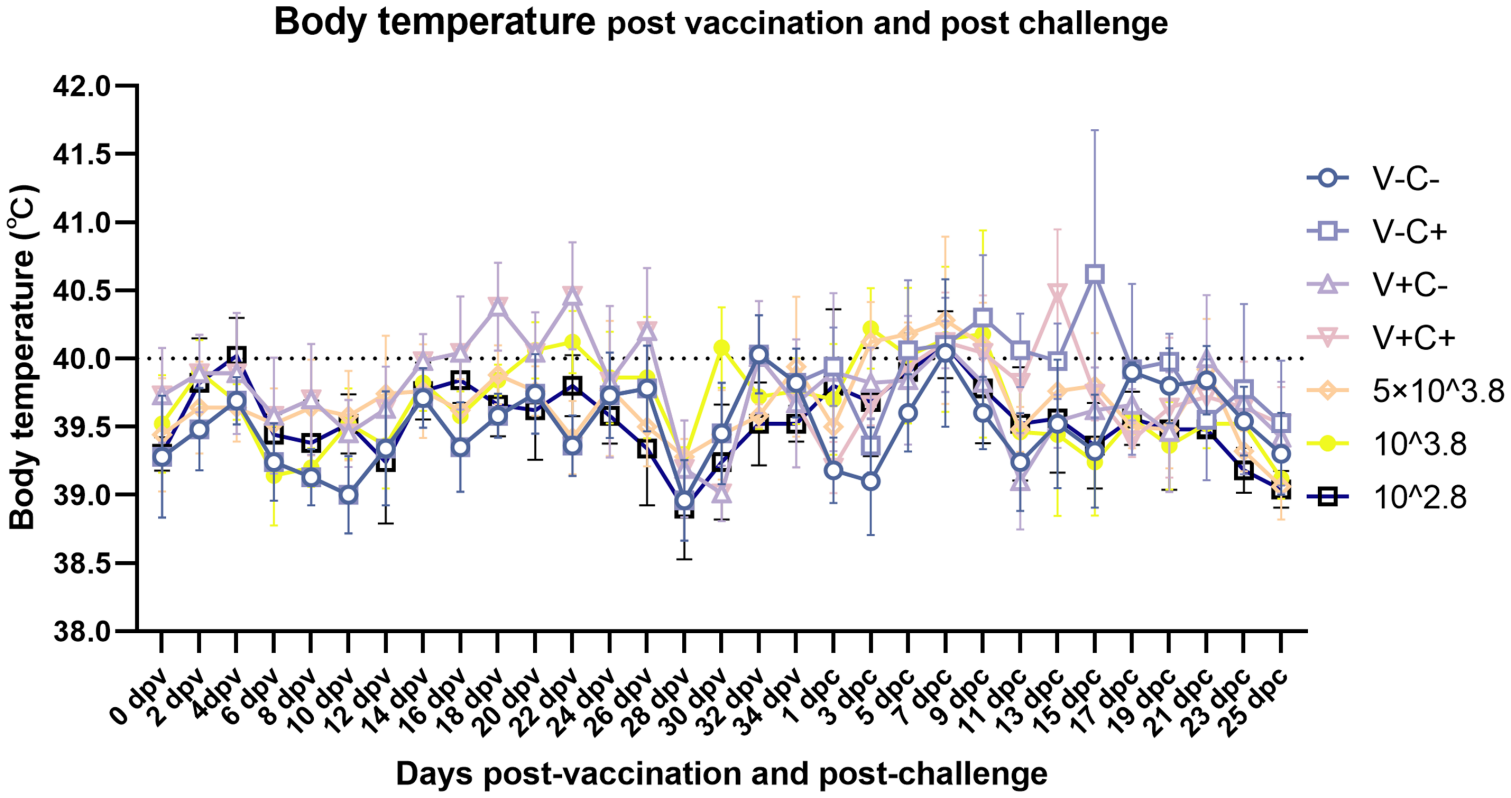
Dynamics of piglet body temperature after vaccination and viral challenge on different dates. Clinical fever was set at 40°C. V ± means groups with the vaccination or not. C ± means groups with the challenge or not. All the reduced-dose groups were challenged. The means ± SDs (error bars) of the temperatures are shown.

**Figure 4:**
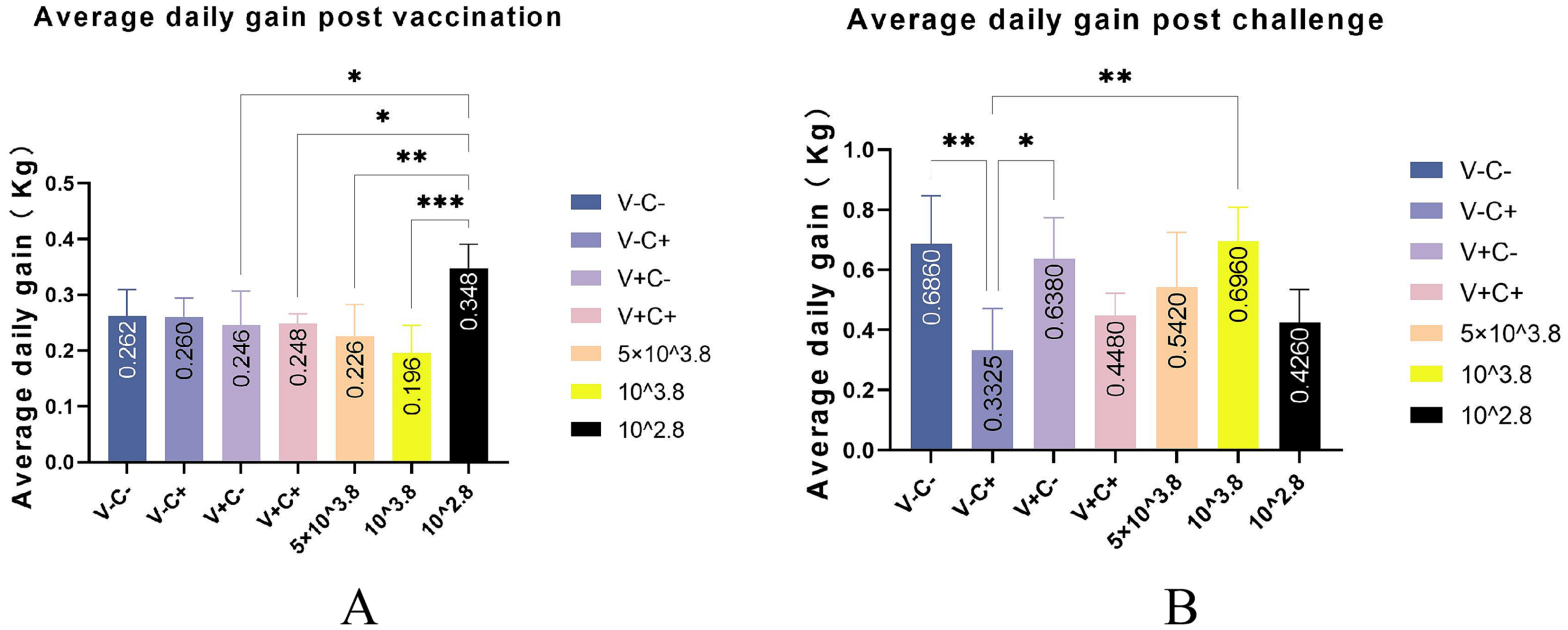
Average daily gain post-vaccination and post-challenge. (A) Average daily gain post-vaccination of different groups. (B) Average daily gain post-challenge of different groups. V ± means groups with the vaccination or not. C ± means groups with the challenge or not. All the reduced-dose groups were challenged. * Indicates a statistically significant difference (*: *p* < 0.05; **: *p* < 0.01; ***: *p* < 0.001).

After vaccination, piglets in the 1 dosage group showed mild symptoms, including a mild fever from 14 to 26 days post-vaccination (dpv), lasting 12 days of that period, and in the dosage reduction group, only two piglets from the 0.01 dosage group showed transient loss of appetite (less active in food, extended eating time, and slower eating speed) at 2 dpv (Figure 2A). From 18 to 22 dpv, the clinical scores of the groups immunized with 0.5 dosage and 0.01 dosage of vaccines were significantly lower than those of the 1 dosage group. Piglets in the 1 dosage group showed obvious fever symptoms, with temperatures reaching up to 41°C from 14 dpv to 26 dpv (after 13 days), and the groups that reduced the dosage did not show significant fever except for the 0.1 dosage group from 20 to 22 dpv (after 3 days) (Figure 3). The average daily gain of the 0.01 dosage group was significantly higher than other immune dosage groups before the challenge (Figure 4A). The low-dosage groups showed higher safety and better growth performance before the challenge.

At 35 dpv, piglets in the phosphate-buffered saline (PBS) group, as well as those receiving the 1 dosage, 0.5 dosage, 0.1 dosage, and 0.01 dosage of the vaccine, were challenged with the NADC30-like PRRSV strain *CF.* According to Figure 2B, the clinical scores of the challenged groups immunized with the 0.5 dosage, 0.1 dosage, and 0.01 dosage of the vaccine were significantly lower than those of the challenge control from 13 to 17 dpc. Piglets in the challenge control group had a fever (up to 41.6°C) from 5 dpc to 19 dpc (after 15 days). In contrast, piglets receiving the 0.5 dosage (up to 41.2°C) and 0.1 dosage (up to 41.3°C) showed fever from 3 dpc to 9 dpc (after 7 days), while those in the 1 dosage group (up to 40.9°C) had a fever from 7 dpc to 13 dpc (after 7 days). Piglets in the 0.01 dosage group (up to 40.7) showed fever only at 7 dpc (Figure 3). Notably, the average daily gain of the 0.1 dosage group was significantly higher than the challenge control (Figure 4B). The data after the challenge still showed that the piglets from the low-dosage group had milder symptoms and better growth performance.

One piglet in the group of challenge control died at 19 dpc, while piglets from other groups survived until the end of the experiment.

To sum up, before the challenge, reducing the dosage appropriately has higher safety and better growth performance of piglets. After the challenge, the low-dosage vaccine can significantly reduce the clinical symptoms and reduce fever temperature and duration caused by the NADC30-like PRRSV *CF* strain to varying degrees. Moreover, the growth performance of piglets vaccinated at a reduced dosage may be better than at 1 dosage after the challenge.

### 3.2 Pathological and histopathological examination

**Figure 5:**
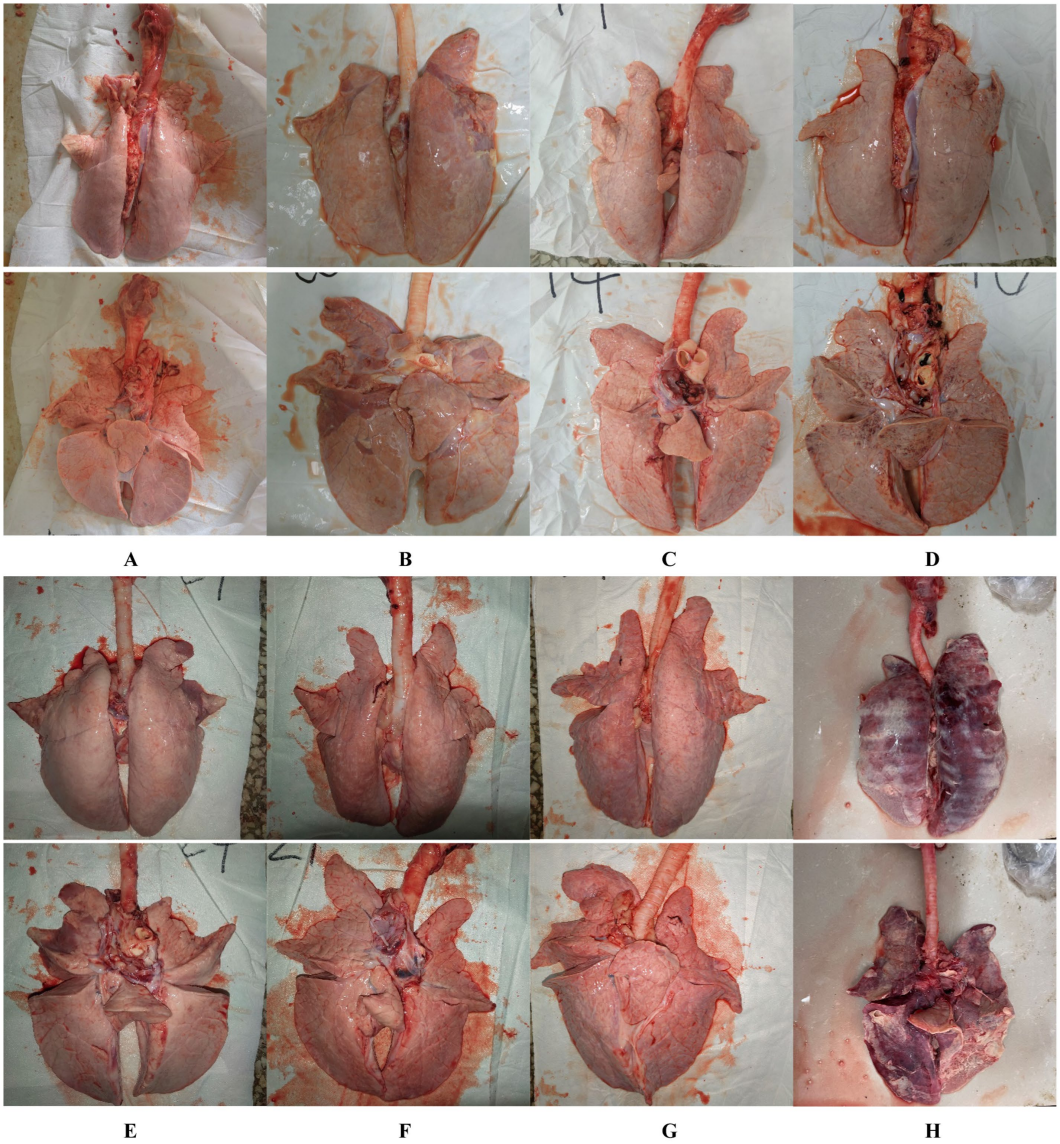
Gross lesions of the lungs from the negative control piglets (A), challenge control piglets (B), piglets immunized without being challenged (C), and piglets immunized with being challenged (D: 1 dosage, E: 0.5 dosage, F: 0.1 dosage, and G: 0.01 dosage). H is the anatomical photo of the dead piglet from the challenge control group.

**Figure 6:**
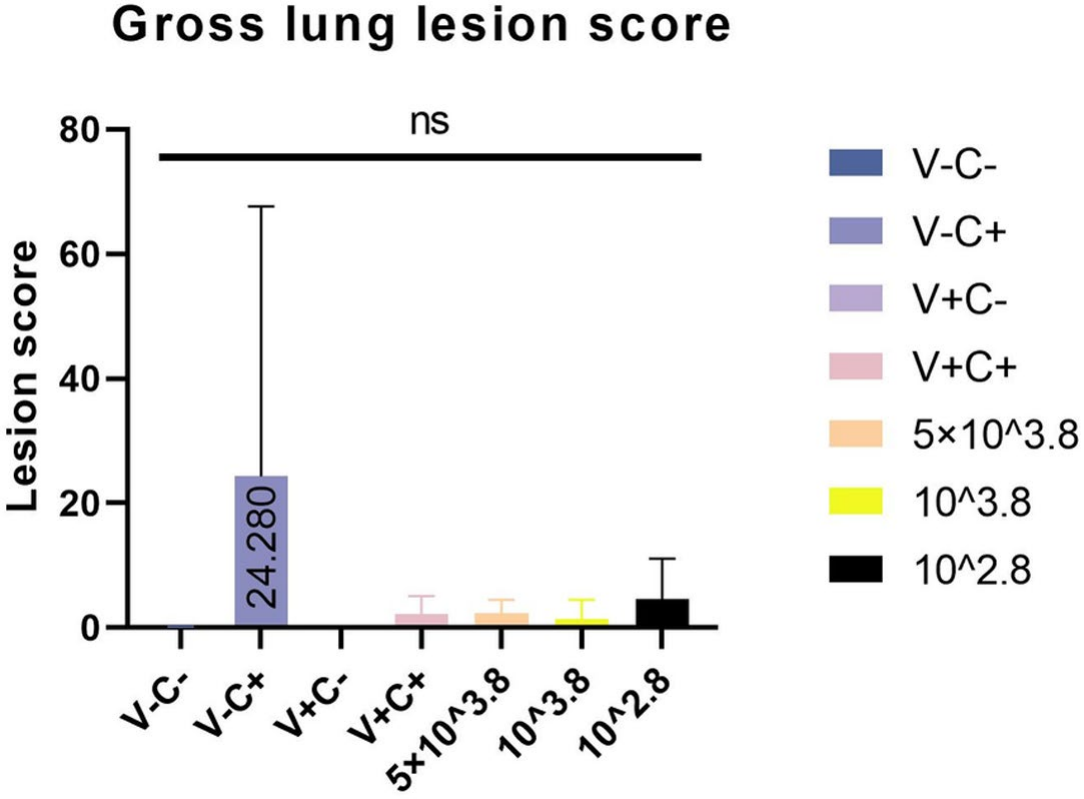
Gross lung lesion score.

**Figure 7:**
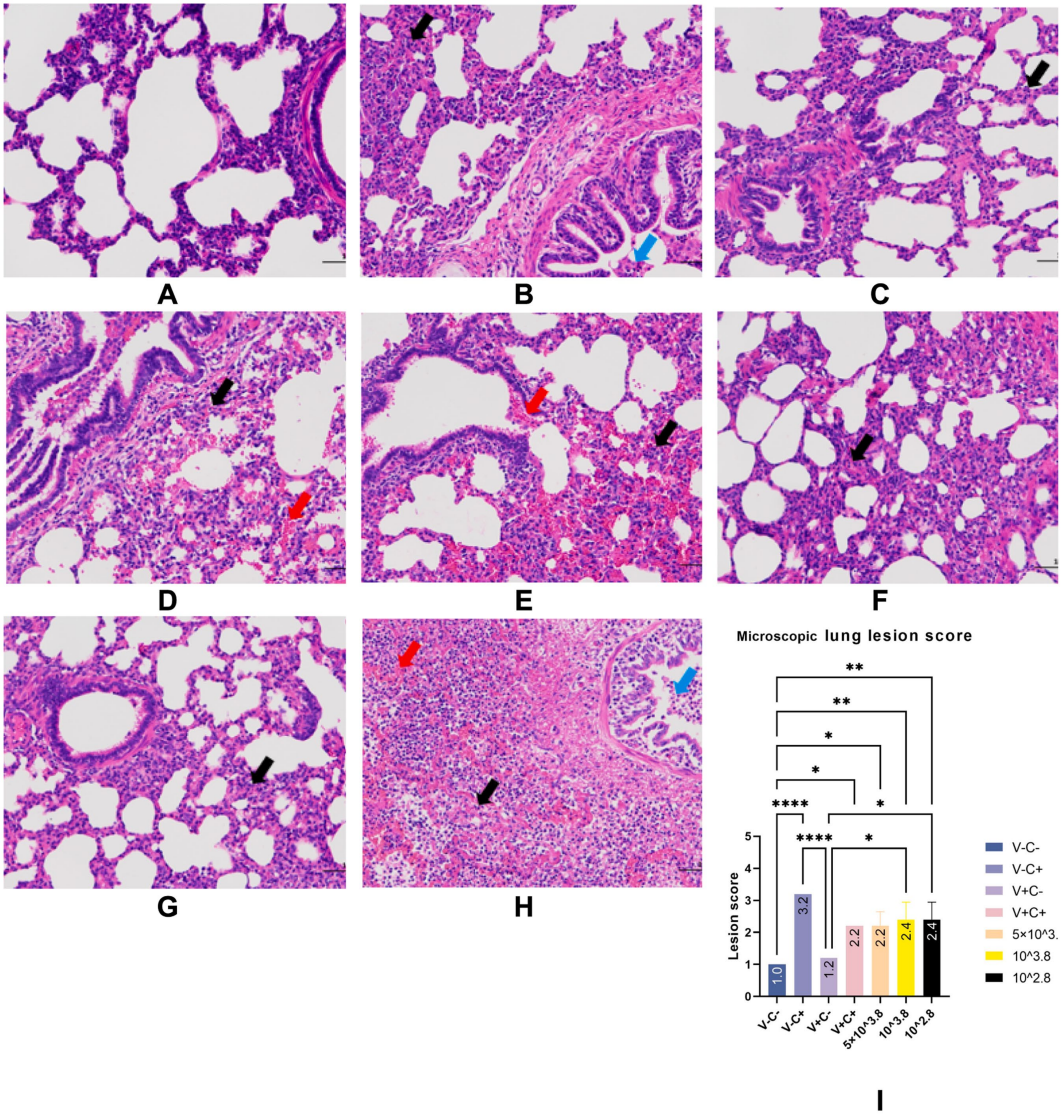
Typical HE manifestations (A–H) and lung tissue section score (I). Groups of negative control piglets (A), challenge control piglets (B), piglets immunized without being challenged (C), and piglets immunized with being challenged (D: 1 dosage, E: 0.5 dosage, F: 0.1 dosage, G: 0.01 dosage). H is the HE manifestation of the dead piglet from the challenge control group. Black arrows indicate inflammatory cell infiltration. Red arrows indicate hemorrhage. Blue arrows indicate necrotic cells. Original magnification, 200 × .

At necropsy, hemorrhage was found in the lungs of the piglets from the challenged 1dosage group (Figure 5D). Obvious lobular pneumonia and lesion boundaries were found in the lungs of the piglets from the challenge control and challenged 0.5 dosage groups (Figures 5B,E). Edema was present in the lungs of piglets in the control group of the attack virus (Figure 5B). The lungs of the dead piglets in the challenged control group had obvious lobar pneumonia and severe cellulosic exudation (Figure 5H), and other groups not described appeared normal (Figures 5A,C,F,G). There was no difference in the score of pulmonary gross lesions among all groups (Figure 6). At histopathological examination (Figures 7A–H), interstitial pneumonia (Interstitial pneumonia was characterized by the thickening of alveolar septa and infiltration of mononuclear cells) was found in the piglets in all immunized and challenged groups, but the challenge control group showed more severe symptoms. The diffuse lesions with large lesion areas were found in the dead piglet of the challenge control group, and bleeding can also be observed (Figures 5, 7H). However, in all vaccinated challenge groups, one or two piglets exhibited moderate diffuse lesions or severe lesion distribution. The lung tissue sections were scored (Figure 7I), revealing that the challenged groups had significantly higher scores than the negative control group. Additionally, except for the 0.5 dosage and 1 dosage groups, the other challenged groups had significantly higher scores than the immune control group.

**Figure 8:**
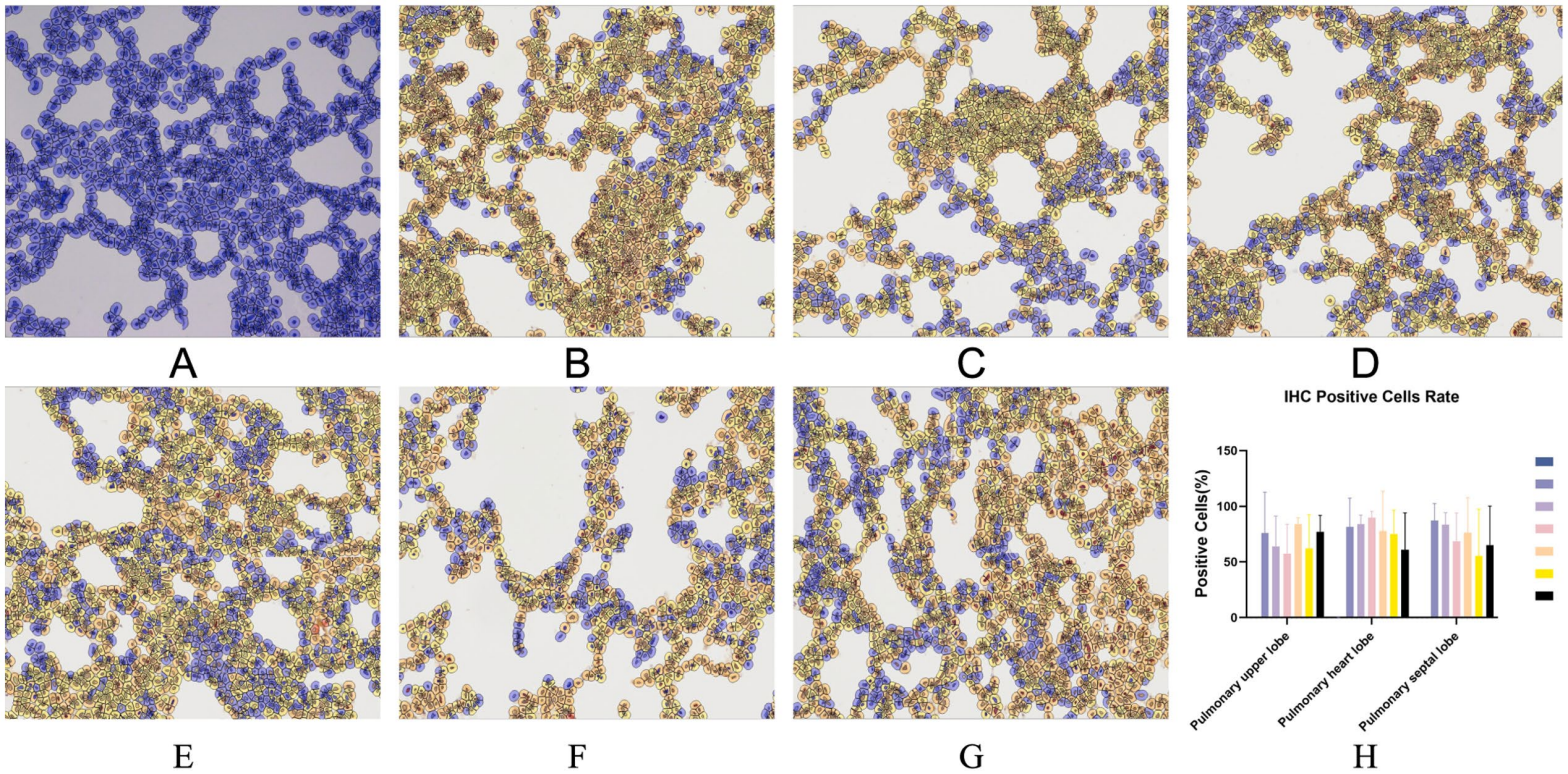
Typical IHC manifestations of negative control piglets **(A)**, challenge control piglets **(B)**, piglets immunized without being challenged **(C)** and piglets immunized with being challenged (**D**: 1 dosage, **E**: 0.5 dosage, **F**: 0.1 dosage, **G**: 0.01 dosage). IHC positive cells rate (**H**). Original magnification, 200×.

Immunohistochemistry (IHC) staining of the lung was also performed to detect the viral antigen. As shown in Figure 8, except for the negative control group, there was no significant difference in cell positivity rates between the remaining six groups. No positive staining was detected in the lungs of piglets in the negative control group.

### 3.3 Viremia examination

**Figure 9:**
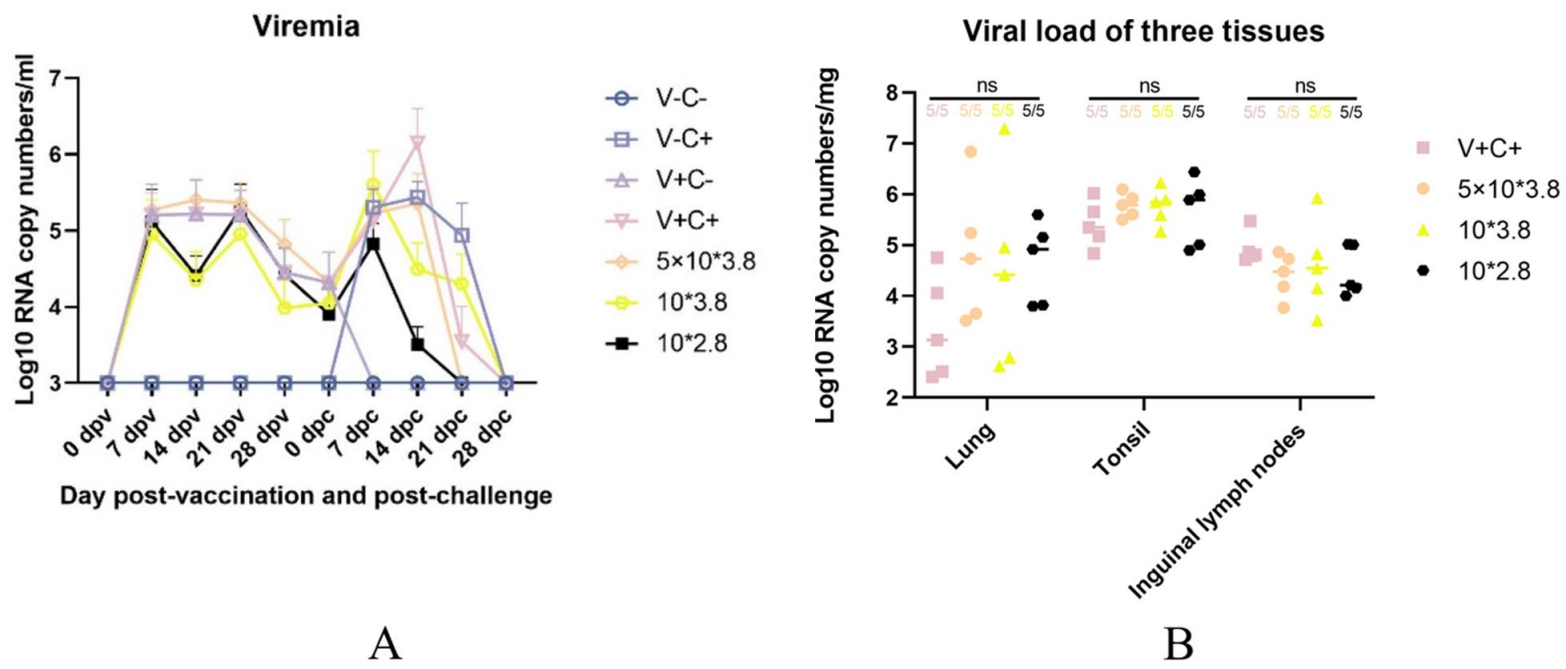
Viremia after vaccination and CF PRRSV challenge and viral load in the three tissues. **(A)** The dynamics of viremia were detected by real-time RT-qPCR. **(B)** Viral loads in different tissues were detected by real-time RT-qPCR. ns: non-statistically significant. V± means groups with the vaccination or not. C± means groups with the challenge or not. All the reduced-dose groups were challenged.

Pig serum samples were taken at 0, 7, 14, 21, 28 dpv and 0, 7, 14, 21, 28 dpc for viremia assessment following the immunization and PRRSV challenge. As shown in Figure 9A, the 1 dosage group had substantially more serum viral RNA copy numbers than the other groups at 14 dpc. There were no significant differences in the virus RNA copy numbers across the groups at other time points. According to Figure 9B, there were also no significant differences in the virus RNA copy numbers among the lung, tonsil, and inguinal lymph nodes of the immunized and challenged groups. The data show that piglets from groups of the low-dosage vaccine had milder viremia than the 1 dosage group.

### 3.4 Serological test

**Figure 10:**
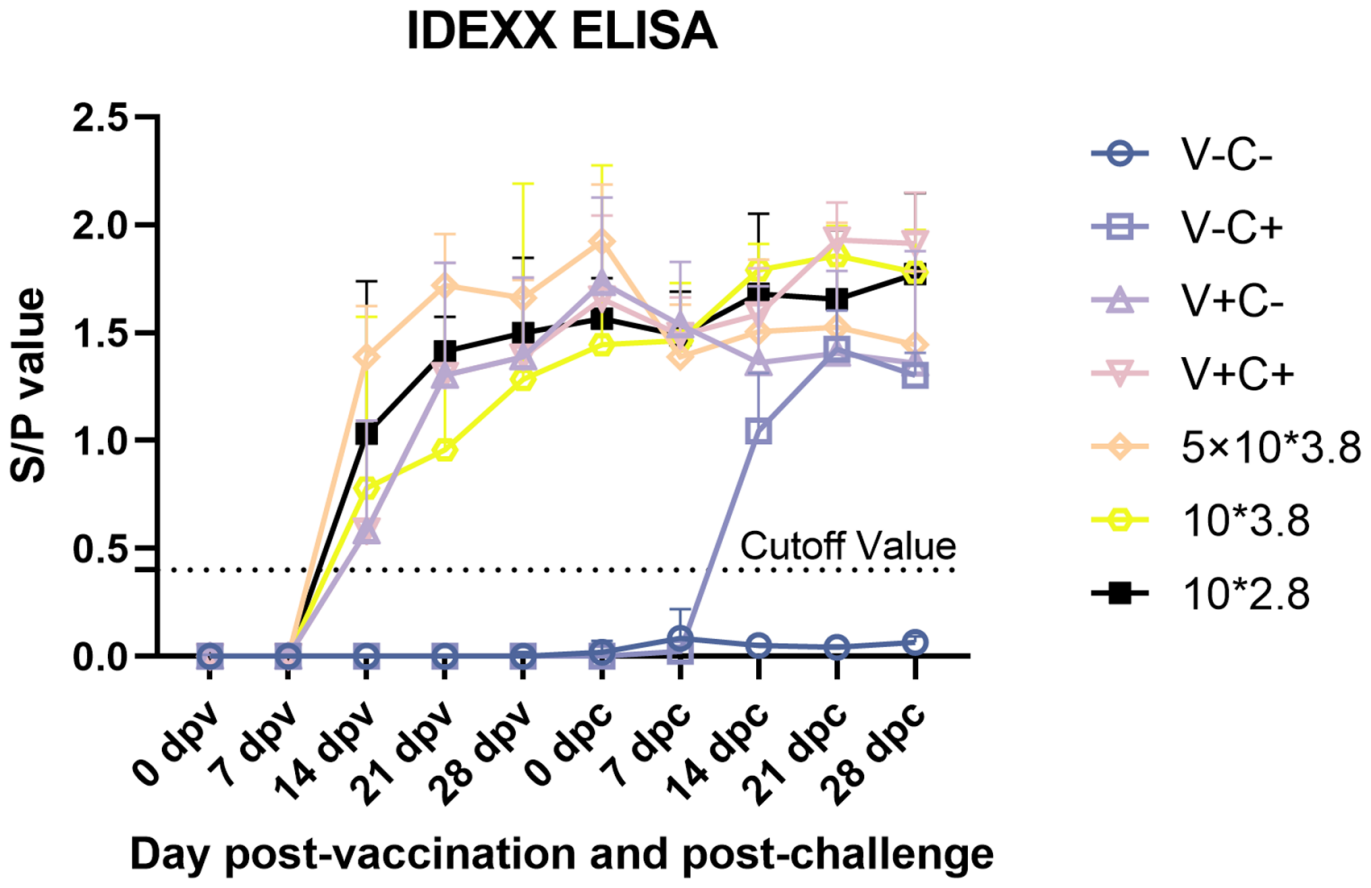
PRRSV-specific antibodies in each group following a PRRSV vaccination or challenge. The threshold for seroconversion was set at a sample-to-positive (s/p) ratio of 0.4, complying with the manufacturer’s guidelines. V ± means groups with the vaccination or not. C ± means groups with the challenge or not. All the reduced-dose groups were challenged. Each bar represents the average for five piglets + SEM.

Following immunization, PRRSV-specific antibodies were assessed using an IDEXX ELISA kit. At 14 dpv, all piglets in the immunized groups exhibited PRRSV-positive antibodies, as depicted in Figure 10. At 14 dpv, the antibody titer of the piglets in the 0.5 dosage group was substantially higher than that of the groups receiving 1 dosage and 0.1 dosage. At 21 dpv, the antibody titer of piglets in the 0.5 dosage group was significantly higher than the 0.1 dosage group. At 28 dpv, there was no discernible variation in antibody titers across the immunization groups. Pigs in the challenge control group had PRRSV-positive antibodies 14 days after the PRRSV challenge, and their antibody titer was substantially lower than that of the pigs in the 0.1 and 0.01 dose groups. At 28 dpc, the antibody titer of piglets in the 1 dosage group was significantly higher than the challenge control group.

Overall, before the challenge, piglets vaccinated with 0.5 dosage showed the highest antibody titer. After the challenge, the speed of early antibody production of piglets vaccinated with 0.1 and 0.01 dosages was faster.

## 4 Discussion

The epidemiological situation of PRRSV in China is extremely complicated, and the biological characteristics are constantly changing with rapid mutation and recombination. Low fidelity during RNA virus replication, recombination events, or random mutations all may lead to the emergence of new PRRSV strains (15, 16). Despite the availability of various commercial vaccines, effective control measures of PRRSV worldwide remain a challenge for pig production. The use of MLV vaccination has been widely accepted in many countries (17, 18). China has made extensive use of five commercial MLV vaccines, but the most widely used is still Ingelvac PRRS MLV from Boehringer Ingelheim, and pig farms have a high consumption rate of this vaccine.

The NADC30-like PRRSV has been prevalent in China since 2013 and has become one of the main epidemic strains (9, 10). In terms of genetics, NADC30-like PRRSVs differ from other PRRSV strains because they contain three discontinuous deletions in the nsp2 gene when compared to VR2332. The reported NADC30-like PRRSVs have nucleotide similarity between 93.5 and 95.7% (14). Partial cross-protection of commercial vaccines against the currently circulating NADC30-like PRRSV may be explained by the low genomic similarity between NADC30-like PRRSV and existing vaccine strains of PRRSV.

The field symptoms of the current circulating strain NADC30-like PRRSV range from low virulence to high virulence (19–22). Although the NADC30-like PRRSV strain is less virulent than the highly pathogenic porcine reproductive and respiratory syndrome virus (HP-PRRSV), it can still lead to small-scale abortions in sows and respiratory diseases in fattening pigs, resulting in significant losses for pig farms (23). At present, there is no commercially available vaccine against the NADC30-like strain of PRRSV in China (24). Some studies have shown that commercially available attenuated vaccines provide a degree of protection against the NADC30-like PRRSV strains, alleviating symptoms and reducing lesions, thereby ensuring piglet survival after a moderately virulent virus attack (14). Many farms consider vaccinating piglets and reducing vaccine doses as appropriate to achieve protective effects while reducing costs. The effectiveness of this dosage reduction against the NADC30-like PRRSV has never been assessed and contrasted, and we would like to know if this is a scientific move. As a result, we evaluated the effectiveness of various vaccine dosages against a NADC30-like PRRSV infection in this study. Fortunately, we found that reducing the dosage can better help piglets mitigate the negative effects of the virus.

In this study, the NADC30-like strain *CF* (PQ213447) was selected from the pool of well-characterized NADC30-like strains from Chengdu SG-Biotech, which showed the highest virulence compared to other strains in the pathogenicity study. After comparison, *CF* shared 93.2, 85.3, and 85.1% nucleotide similarity with NADC30 (JN654459), VR2332 (AY150564), and HP-PRRSV representative strain JXA1 (EF112445), respectively. Following the viral challenge, piglets that had not received vaccinations had exhibited typical PRRSV clinical signs. There was one dead piglet in the challenge control group during the study; we think that its death may not only be the cause of the PRRSV virus, but the PRRSV challenge may have contributed to its death.

When comparing average daily gain, we found that the reduced-dosage groups outperformed the other groups before or after the challenge (Figures 4A,B). However, due to the limitation of the experimental site, it may not be 100% free feeding, and the results are for reference only. Piglets in all challenged groups had viremia that peaked at 7 and 14 dpc (Figure 9A). At the end of the study, there was no significant difference in the amount of virus RNA copies across the challenged groups, even though the virus titers continued to decline after that. All of the vaccinated and challenged piglets showed comparable levels of viral antigen in various tissues, which was consistent with the viremia results. There are individual differences in viremia, for reference only. Humoral immunity is considered to play a significant role in protecting pigs from PRRSV infection (18). Measurements of virus-neutralizing (VN) antibodies and PRRSV-specific antibodies were made to investigate the humoral immunity brought on by vaccination. Unfortunately, due to problems with sample preservation and repeated freeze–thaw, the neutralizing antibody results that we obtained may not be reliable and are not presented here. At 14 days post-vaccination, all vaccinated groups developed positive PRRSV-specific IDEXX ELISA antibodies (Figure 10).

In the comparison of clinical scores and body temperature changes, reduced-dosage vaccinated piglets had fewer side effects before the challenge and better performance after the challenge (Figures 2, 3). There were no significant differences among the vaccinated challenge groups in terms of gross and section scores or the rate of positive cells (Figures 6–8). In Figure 10, we saw that the specific antibodies in the reduced-dosage group were higher than those in the original dosage group before the challenge.

In conclusion, low-dosage MLV vaccine immunization before the challenge has fewer adverse side effects on the piglets and therefore has better growth performance. After the challenge, the low-dosage vaccine was able to reduce the symptoms caused by the NADC30-like PRRSV, with some aspects showing even better relief effects than the normal dosage. The specific mechanism behind this phenomenon is intriguing, and further research is needed to uncover it. Further considering the actual production, reducing the vaccine dose also means reducing the cost. Until an effective vaccine against the NADC30-like PRRSV strain is available or a better vaccine program is discovered, it may be beneficial to reconsider vaccine doses. This approach could enhance economic value while also focusing on determining the immune target, understanding the virulence of the virus in the field, and ensuring safety.

## Data Availability

The datasets presented in this study can be found in online repositories. The names of the repository/repositories and accession number(s) can be found in the article/supplementary material.
